# Do electronic and economic empowerment protect women from intimate partner violence (IPV) in India?

**DOI:** 10.1186/s12905-022-02110-4

**Published:** 2022-12-09

**Authors:** Koustuv Dalal, Masuma Yasmin, Heléne Dahlqvist, Gunnar O. Klein

**Affiliations:** 1 Division of Public Health Science, School of Health Sciences, Mid Sweden University, Sundsvall, Sweden; 2 Kolkata, India; 3 Informatics Section, Centre for Empirical Studies on Information Systems, School of Business, Örebro University, Örebro, Sweden

**Keywords:** Domestic violence, Economic empowerment, Electronic empowerment, India

## Abstract

**Background:**

Intimate partner violence (IPV) is a major public health problem. Electronic empowerment has several positive impacts on health. No study has examined whether electronic empowerment prevents intimate partner violence. Economic empowerment has positive and negative effects on IPV victimization. The current study was conducted to investigate whether economic and electronic empowerment of women act as protective factors against IPV in India.

**Methods:**

A national representative sample of 66,013 ever-married women from 36 member states and union territories of India has been used from the National Family Health Survey 2015 to 2016. Emotional, physical and sexual violence against women by husbands were target variables. We used bivariate and multivariate analyses.

**Results:**

The prevalence of emotional violence was 13%, physical violence was 28% and sexual violence was 7%. IPV against women was as follows: The prevalence was higher among women living in rural areas, belonging to Hindu religion and those belonging to Scheduled Castes. Higher education and higher socio-economic status were found to be protective factors against IPV. The prevalence of IPV was higher among the working women, among those having knowledge of business loans for women and the recipients of such business loans. Exposure to media was found to reduce IPV. The women who used mobile phones and SMS facility experienced less violence.

**Conclusion:**

Economic independence of women was found to be a risk factor for IPV in India, whereas electronic empowerment was a protective factor. In the Indian context, policymakers should make use of mobile phones and support SMS use in the IPV awareness programs. Women empowerment, combined with gender equity, can reduce the prevalence of violence against women.

## Background

Intimate partner violence (IPV), in the form of emotional, physical and sexual violence is a major public health problem. IPV affects all countries and ethnicities. Low-and middle- income countries are highly affected by IPV [1–5]. It has several long-term consequences on the physical, sexual-reproductive and mental health of the victims [1, 6]. A plethora of studies have been conducted focusing on IPV and its consequences [7–11]. Theories have explained a series of causes of IPV, such as men’s pathology including abnormal personality traits and/or alcoholism, power relation, cultural norms and institutional practices [12–16]. However, literature has indicated that no single theory is able to provide sufficient empirical support to controlling the entire IPV phenomena [12, 13].

Various economic empowerment strategies, such as, microfinance, group savings and livelihood efforts have been undertaken in order to provide women with financial security and independence, aiming to alleviate the prevalence of IPV [15, 17]. But these methods do not take into account the deep-rooted gender biases that exist in the Indian society and therefore, these approaches are not enough to reduce violence against women. It is often thought that empowering women economically will improve their status in the society as well as in the family, thereby giving them the decision-making power and the freedom to leave abusive relationships [15, 18, 19]. It also results in improvement of help-seeking behavior among the victims of violence [20]. However, it is widely observed that financially-independent women stand a higher risk of being abused and victimized, because their higher socio-economic status goes against the patriarchal society and hurts the male ego [15–18, 21].

In its Beijing declaration, the United Nations has strongly endorsed ‘economic empowerment’ of women as a protective factor for IPV against women [5, 12, 22]. However, studies have indicated that the reverse may be true [18]. Some studies have supported the Beijing declaration and advocated that women economic empowerment generates more economic resources for the women and family, resulting in a decrease of IPV [13], while other studies have argued against it [12]. They have propounded that IPV will increase as husbands make effort to compensate for enhanced women status and their economic independence due to empowerment. A Previous study from India indicated that the working women in families where female are the head-of the family were more exposed to IPV than male headed families. The reason could be that women are viewed as inferior due to the patriarchal nature of Indian society, and they are always dependent on the men in the family. The cultural belief is that men are supposed to go out and earn money, whereas women are supposed to stay at home and do household work. The study suggested the inclusion of household work, care work and other voluntary work into the national income account, in order to change this notion [12]. A study from South Africa indicated that economic empowerment through microfinance has reduced the prevalence of IPV around 50% [13].

Economic independence of women alone cannot bring about a remarkable change in the prevalence of IPV against them. A multitude of other factors, such as, gender equity, status and position of women in society, cultural and gender norms of countries, female education, electronic empowerment and social participation determine the vulnerability of women to abuse and violence. Most often women are victimized by their husbands or other intimate partners and it usually occurs due to the innate gender bias that exists in low and middle income countries. Therefore, in order to achieve a drastic reduction in prevalence of IPV against women, all these factors must be taken into account [12, 18].

Exposure to media also has a significant relation to IPV [23]. Electronic (or E-) empowerment is obtained from participation in any online or e-enabled context. Studies have indicated that participation increases influence and control resulting in enhanced empowerment [24]. Therefore e-empowerment has close relation with influence and control, which could be a protective factor for the women against domestic violence. Moreover, exposure to media increases the help-seeking behavior among the women, which ultimately, results in reduction of IPV against them [20]. However, no study has tried to assess whether e-empowerment prevent IPV.

In India, like in other low- and middle-income countries, there is a high prevalence of violence against women [5, 25]. Therefore, the current study had been undertaken to investigate whether economic and electronic empowerment protects women in India from IPV, through a nationally representative sample in 29 states and seven union territories. The study also investigated whether exposure to media protects women from IPV victimization.

## Methods

This was across-sectional study using the National Family Health Surveys (NFHS)-4 from 2015 to 2016. The Government of India commenced the National Family Health Surveys (NFHS) to generate reliable quality data on demographic, socio-economic and various health indicators. In collaboration with the Measure DHS, NFHS-1 (1992–1993), NFHS-2 (1998–1999), NFHS-3 (2005–2006) and the NFHS-4 (2015–2016) was conducted. The NFHS-4 was intended to support policy makers in the health sector, and for the purposes of the current study, the NFHS-4 that also highlights domestic violence against women, has been used.

### NFHS-4 sampling and data collection

NFHS-4 used two stages and three stages sampling techniques for the rural and urban areas, respectively based on 2011 population census. In the rural areas, first, using probability proportional to size (PPS), villages were chosen as primary sampling units (PSUs) from where households were randomly selected. In the urban areas, first, using PPS, municipality wards were selected as PSUs from where census enumeration blocks (CEB) were randomly selected. At the next and final stage, households were randomly selected from each CEB.

Fieldwork was conducted in two phases from January 2015 to 4 December 2016. In total 789 trained filed teams of seven members consisting of three female and one male interviewers, two health investigators led by a field supervisor and the driver, collected the data from 28,522 clusters in all over India.

NFHS-4 initially selected 628,900 households for sampling. However, 616,346 households were occupied. From the occupied households, 601,509 were finally successfully interviewed. From those households, with a 97% response rate, a total of 699,686 women of reproductive age (15–49 years) were interviewed for the NFHS questionnaires. A completed explanation of the entire data collection procedure including sampling methods is available elsewhere [26].

However, to comply with the ethical conditions, from each selected household, only one eligible woman was randomly selected for responding to IPV questions.

### Questionnaire

The questionnaires of NFHS-4 provide data regarding women’s demographic and socio-economic background, empowerment and domestic violence.

### Variables

Among all the 699,686 women of reproductive age (15–49 years), the current study included the ever married women who had ever experienced any IPV (N = 66,013).

IPV questionnaires were developed based on consultation with several experts and survey research on domestic violence measurement [27]. The IPV questions were originally based on a modified version of the Conflict Tactics Scale [28]. The IPV questions were focused on ever respondent’s (15–49 years) exposures to physical, emotional, and sexual violence in the domestic arena.

### Dependent variables

In the current study we consider Intimate partner violence (IPV) with exposure to one or several of the emotional, physical and sexual violence, as defined below [26–28]:

- Emotional violence: husband ever humiliated and/or threatened her with harm and/or insulted her to feel bad.
- Physical violence: husband ever (anyone or combination) pushed, shook, slapped, punched, kicked, dragged or threw something. Also, husband ever tried to strangle or burn and/or attacked with any weapons.
- Sexual violence: husband ever physically forced to penetrate sex or sexual performance when not wanted.

### Independent variables

Demographic characteristics include age (seven groups: 15–19, 20–24, 25–29, 30–34, 35–39, 40–44 and 45–49); residency (rural/urban); education (No education, primary, secondary and higher); religion, caste (scheduled castes (SC), scheduled tribes (ST), other backward classes (OBC) and others) and economic status. Religion consisted of Hindu, Muslim, Christian, Sikh, Buddhist, Jain, Jewish, Parsi and no religion. However, during the analysis it was found that apart from the first two religions (i.e. Hindu and Muslims), women from other religions responded at very low rates to the IPV questionnaire. The current study has merged all other religions and formed the new heading ‘others’.

Economic status was measured by the Wealth index, a composite measure of the household’s cumulative standard of living. The Wealth index is mainly used to measure the ability of the respondents to pay for health services and also the distribution of the services among the poor. For calculation of Wealth index, the data on ownership of household assets such as, radio, television and bicycle, materials used for construction of house, type of water supply, and sanitation facilities are used. A statistical method known as the principal components analysis is used, which puts individual households on a continuous scale of relative wealth (standard normal distribution: Mean = 0 and standard deviation = 1). Finally, different categories of wealth quintiles are established using the standardized scores, such as, poorest, poorer, middle, richer and richest. In India, the Wealth index was introduced by Rutstein and Johnson (2004), which includes the items indicating economic status [12, 29].

Respondent’s working status was very important to measure the economic empowerment of women. The working status was indicated by whether the respondent was working or not [12]. Bank account operated by the respondent; knowledge of business loans for women and recipient of startup/expansion business loan were also included in the study as part of economic empowerment.

Empowerment variables, including exposure to media were important in the analysis, as per the objectives [23]. Exposure to media consists of reading newspapers/magazine; listening to radio; and watching TV. Exposure to media had four options: no exposure, less than once a week, at least once a week and almost every day. In addition to this, going to cinema hall or theatre (yes/no) was included in the study. All these variables are important as exposure to media in LMIC context which has emerged as protective factors [23].

Respondent’s use of mobile phone and if she was using SMS (Short Message Service) to create and receive text messages were included in the study for assessing their electronic empowerment (E-empowerment).

### Statistical analysis

Chi-square tests were used to examine differences in proportions of exposure to IPV by demographic, socioeconomic and empowerment variables. Multivariate logistic regression analysis was performed with all demographic, socioeconomic and empowerment (including electronic) variables to assess their independent contribution in predicting exposure to IPV. IBM SPSS v27 was used for analysis. Statistical significance was studied at P < 0.05.

## Results

Among 66,013 women respondents of reproductive age, 13% faced emotional violence, 28% physical violence and 7% experienced sexual violence performed by their husbands.

The women residing in rural areas experienced more IPV than urban women (emotional violence: 13% vs. 12%, physical violence: 30% vs. 24%, and sexual violence: 7% vs. 5%, respectively). The Hindu women experienced more physical (30%) and sexual (7%) IPV than women from the Muslim community (physical violence; 22%, sexual violence; 6%) or women of other religions in India (Table 1). Scheduled Caste women experienced higher prevalence of IPV (emotional violence; 16%, physical violence; 35%, sexual violence; 9%), than general caste or other caste women. Higher education of the respondents demonstrates less exposure to IPV. In addition, the women belonging to the richest wealth quintile experienced less violence (Table 1).

**Table 1:** Number of Women in Each Category (N) and Proportion within Each Category Exposed to Intimate Partner Violence (Percentage of N), in terms of Socio-demographic Characteristics

	N	Emotional violence	Physical violence	Sexual violence
*Age*		P < 0.001	P < 0.001	P = 0.272
15–19	1642	197 (12.0%)	333 (20.3%)	107 (6.5%)
20–24	8847	1002 (11.3%)	2309 (26.1%)	605 (6.8%)
25–29	13,970	1700 (12.2%)	3829 (27.4%)	931 (6.7%)
30–34	13,598	1740 (12.8%)	4039 (29.7%)	943 (6.9%)
35–39	11,402	1496 (13.1%)	3293 (28.9%)	749 (6.6%)
40–44	8677	1161 (13.4%)	2543 (29.3%)	561 (6.5%)
45–49	7877	1076(13.7%)	2334 (29.6%)	476 (6.0%)
*Residence*		P < 0.001	P < 0.001	P < 0.001
Urban	19,469	2254 (11.6%)	4745 (24.4%)	1059 (5.4%)
Rural	46,544	6118 (13.1%)	13,935 (29.9%)	3313 (7.1%)
*Religion*		P < 0.001	P < 0.001	P < 0.001
Hindu	49,546	6449 (13%)	14,818 (29.9%)	3409 (6.9%)
Muslim	8614	1106 (12.8%)	2082 (22.4%)	510 (5.9%)
Others	7853	817 (10.4%)	1780 (22.7%)	453 (5.8%)
*Caste*		P < 0.001	P < 0.001	P < 0.001
Schedule caste	11,686	1843 (15.8%)	4127 (35.3%)	1040 (8.9%)
Schedule tribe	12,108	1549 (12.8%)	3407 (28.1%)	824 (6.8%
Other backward class	25,574	3390 (13.3%)	7801 (30.5%)	1714 (6.7%)
Others	13,719	1223 (8.9%)	2814 (20.5%)	648 (4.7%)
*Education*		P < 0.001	P < 0.001	P < 0.001
No-education	22,028	3630 (16.5%)	8181 (37.1%)	1928 (8.8%)
Primary	9669	1371 (14.2%)	3210 (33.2%)	754 (7.8%)
Secondary	28,187	2994 (10.6%)	6508 (23.1%)	1522 (5.4%)
Higher	6129	377 (6.2%)	781 (12.7%)	168 (2.7%)
*Economic status*		P < 0.001	P < 0.001	P < 0.001
Poorest	12,838	2254 (17.6%)	5267 (41%)	1353 (10.5%)
Poorer	13,992	2066 (14.8%)	4632 (33.1%)	1075 (7.7%)
Middle	13,790	1815 (13.2%)	3844 (27.9%)	905 (6.6%)
Richer	13,142	1361 (10.4%)	3042 (23.1%)	645 (4.9%)
Richest	12,251	876 (7.2%)	1895 (15.5%)	394 (3.2%)

Χ^2^ significance level

The women who did not read newspapers or magazines at all were almost two times more exposed to IPV than the women who read almost every day. The women who did not watch television were more exposed to IPV than their peers who watched television frequently (emotional violence: 15 vs. 11%, physical violence: 35% vs. 24% and sexual violence: 9% vs. 5%, respectively). Similarly, the women who went to the cinema or theatre at least once a month were less exposed to IPV. Working women were more exposed to IPV than their unemployed peers (emotional violence: 17% vs. 11%, physical violence: 36% vs. 26%, and sexual violence: 9% vs. 6%, respectively). The respondents having knowledge of business loans for women experienced more emotional (13%) and physical violence (30%). Moreover, the recipients of start-up or expansion business loan were more exposed to all three types of IPV (emotional violence; 18%, physical violence; 39%, sexual violence; 9%). However, the women who operated bank accounts experienced less IPV than the women who did not operate bank accounts (Table 2).

**Table 2:** Number of women in each category (N) and proportion within each category exposed to Intimate Partner Violence (percentage of N), in terms of empowerment variables (including exposure to media)

	N	Emotional violence	Physical violence	Sexual violence
*Read newspaper/magazine*		P < 0.001	P < 0.001	P < 0.001
Not at all	43,879	6279 (14.3%)	14,199 (32.4%)	3329 (7.6%)
Less than once a week	8663	928 (10.7%)	2062 (23.8%)	442 (5.1%)
At least once a week	6522	626 (9.6%)	1324 (20.3%)	329 (5.0%)
Almost every day	6049	539 (7.8%)	1095 (15.8%)	272 (3.9%)
*Listening to radio*		P = 0.169	P < 0.001	P = 0.026
Not at all	56,326	7131 (12.7%)	16,089 (28.6%)	3680 (6.5%)
Less than once a week	3546	488 (13.8%)	1076 (30.3%)	259 (7.3%)
At least once a week	3565	430 (12.1%)	892 (25.0%)	232 (6.5%)
Almost every day	2576	323 (12.5%)	623 (24.2%)	201 (7.8%)
*Watching TV*		P < 0.001	P < 0.001	P < 0.001
Not at all	16,820	2584 (15.4%)	5864 (34.9%)	1461 (8.7%)
Less than once a week	5067	755 (14.9%)	1780 (35.1%)	409 (8.1%)
At least once a week	7399	974 (13.2%)	2156 (29.1%)	534 (7.2%)
Almost every day	36,727	4059 (11.1%)	8880 (24.2%)	1968 (5.4%)
*Go to cinema/theatre at least once a month*		P = 0.001	P < 0.001	P = 0.004
No	62,142	7945 (12.8%)	17,852 (28.7%)	4159 (6.7%)
Yes	3871	427 (11%)	828 (21.4%)	213 (5.5%)
*Currently working*		P < 0.001	P < 0.001	P < 0.001
No	49,335	5509 (11.2%)	12,709 (25.8%)	2858 (5.8%)
Yes	16,658	2863 (17.2%)	5971 (35.8%)	1514 (9.1%)
*Bank account operated by the respondent*		P < 0.001	P < 0.001	P < 0.001
No	30,272	4081(13.5%)	9218 (30.5%)	2311 (7.6%)
Yes	35,741	4291 (12.0%)	9462 (26.5%)	2061 (5.8%)
*Knowledge of business loans for women*		P = 0.001	P < 0.001	P = 0.576
No	40,187	4960 (12.3%)	10,973 (27.3%)	2679 (6.7%)
Yes	25,826	3412 (13.2%)	7707 (29.8%)	1693 (6.6%)
*Recipient of startup/expansion business loan*		P < 0.001	P < 0.001	P < 0.001
No	20,955	2558 (12.2%)	5830 (27.8%)	1244 (5.9%)
Yes	4871	854 (17.5%)	1877 (38.5%)	449 (9.2%)
*Respondent use mobile phone*		P < 0.001	P < 0.001	P < 0.001
No	32,844	4772 (14.5%)	11,103 (33.8%)	3563 (7.8%)
Yes	33,169	3600 (10.9%)	7577 (22.8%)	1809 (5.5%)
*Able to use SMS*		P < 0.001	P < 0.001	P < 0.001
No	11,021	1731 (15.7%)	3547 (32.2%)	877 (8.0%)
yes	20,823	1676 (8.0%)	3630 (17.4%)	831 (4.0%)

$$\chi^{2}$$ significance level

In addition, the women who used mobile phones (emotional violence; 11%, physical violence; 23%, sexual violence; 6%) were less exposed to all the three types of IPV. Similarly, the women who were able to use SMS experienced almost two times less IPV than their peers who were unable to use the same (Table 2).

**Fig. 1:**
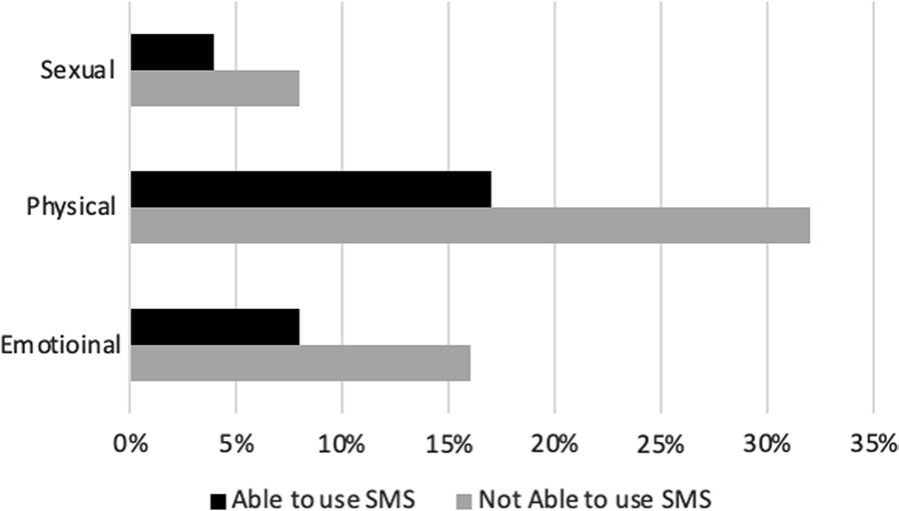
Exposure of women to intimate partner violence, according to their ability to use of sms

Figure 1 presents exposure of women to all three forms of IPV according to their ability to use of sms. Women, who cannot use sms are more exposed to all three forms of IPV.

**Fig. 2:**
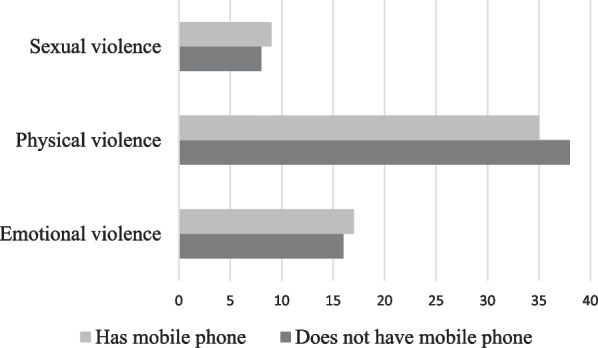
Exposure of illiterate women to intimate partner violence, according to possession of mobile phone

The women who had no education but used mobile phones experienced higher proportions of both emotional and sexual violence, and slightly lower proportions of physical violence (Fig. 2).

Multivariate logistic regression indicates that compared to the rural women, the urban women are more likely to experience IPV, as denoted by the adjusted Odds Ratios (emotional violence; OR: 1.4, CI 1.23–1.59, physical violence; OR: 1.35, CI 1.23–1.49). The women belonging to Scheduled Caste and Other Backward Classes are more likely to be exposed to both emotional and physical violence, as compared to their peers belonging to other castes. When compared to the women having higher education, the illiterate women are about 1.5 times more likely to face both emotional and physical violence, and almost 3 times more likely to face sexual violence. Similarly, the women belonging to the poorest wealth quintile are almost 3 times more likely to be exposed to all the three types of IPV than the richest women (Table 3).

**Table 3:** Adjusted odds ratio (OR) of each category for exposure to intimate partner violence, and confidence intervals (CIs) of OR, in terms of socio-demographic and empowerment variables

	Emotional violence			Physical violence			Sexual violence		
	OR	95% CI		OR	95% CI		OR	95% CI	
*Age*									
15–19	0.625	0.361	1.082	0.548	0.369	0.815*	1.312	0.712	2.420
20–24	0.783	0.610	1.006	0.836	0.694	1.007	1.079	0.77	1.513
25–29	0.861	0.698	1.062	0.853	0.727	1.002	1.052	0.787	1.406
30–34	0.867	0.709	1.060	1.013	0.869	1.181	1.025	0.776	1.355
35–39	0.899	0.732	1.102	0.943	0.806	1.104	0.930	0.697	1.239
40–44	1.032	0.830	1.283	0.995	0.839	1.180	0.926	0.676	1.267
45–49	Ref			Ref			Ref		
*Residence*									
Urban	1.400	1.231	1.593***	1.350	1.226	1.487***	1.295	1.083	1.548*
Rural	Ref			Ref			Ref		
*Religion*									
Hindu	0.924	0.767	1.113	0.910	0.793	1.045	0.719	0.564	0.917*
Muslim	1.020	0.792	1.315	0.818	0.676	0.991	0.735	0.522	1.034
Others	Ref			Ref			Ref		
*Caste*									
Schedule caste	1.521	1.269	1.822***	1.502	1.314	1.717***	1.185	0.932	1.505
Schedule tribe	1.051	0.850	1.300	0.821	0.701	0.962*	0.778	0.587	1.031
OBC	1.355	1.167	1.573***	1.386	1.245	1.543***	0.937	0.766	1.147
Others	Ref			Ref			Ref		
*Education*									
No-education	1.552	1.183	2.038***	1.776	1.450	2.175***	2.785	1.883	4.121***
Primary	1.591	1.212	2.089***	1.728	1.411	2.117***	2.131	1.429	3.179***
Secondary	1.329	1.097	1.609*	1.434	1.251	1.644***	1.869	1.395	2.504***
Higher	Ref			Ref			Ref		
*Economic status*									
Poorest	2.562	1.924	3.413***	3.394	2.737	4.207***	3.605	2.469	5.263***
Poorer	2.666	2.125	3.345***	2.561	2.163	3.033***	2.610	1.898	3.590***
Middle	1.994	1.642	2.422***	1.873	1.626	2.158***	2.062	1.562	2.722***
Richer	1.518	1.278	1.802***	1.549	1.370	1.751***	1.646	1.282	2.114***
Richest	Ref			Ref			Ref		
*Read newspaper/magazine*									
Not at all	0.898	0.741	1.089	1.141	0.989	1.317	0.723	0.553	0.945
< Once a week	0.951	0.779	1.160	1.288	1.113	1.491***	0.719	0.542	0.953
Once a week	0.973	0.793	1.192	1.198	1.032	1.392	0.866	0.653	1.148
Almost daily	Ref			Ref			Ref		
*Listening to radio*									
Not at all	0.996	0.775	1.279	0.895	0.745	1.076	0.564	0.421	0.754
< Once a week	1.103	0.794	1.533	1.189	0.935	1.511	0.675	0.448	1.017
Once a week	0.968	0.692	1.353	0.990	0.775	1.264	0.707	0.472	1.058
Almost daily	Ref			Ref			Ref		
*Watching TV*									
Not at all	1.018	0.838	1.237	1.031	0.886	1.199	1.086	0.839	1.404
< Once a week	0.873	0.671	1.137	1.041	0.858	1.262	1.026	0.733	1.436
Once a week	0.944	0.776	1.149	0.937	0.809	1.085	1.176	0.914	1.515
Almost daily	Ref			Ref			Ref		
*Go to cinema at least once a month*									
No	Ref			Ref			Ref		
Yes	1.135	0.931	1.384	1.167	1.007	1.351*	1.024	0.769	1.363
*Currently working*									
No	Ref			Ref			Ref		
Yes	1.420	1.260	1.601***	1.443	1.316	1.582***	1.470	1.248	1.732***
*Bank account operated by the respondent*									
No	Ref			Ref			Ref		
Yes	1.074	0.940	1.228	0.977	0.885	1.077	0.927	0.777	1.104
*Knowledge of business loans for women*									
No	Ref			Ref			Ref		
Yes	1.370	1.201	1.562	1.626	1.524	1.736***	1.609	1.437	1.801***
*Taken startup/expansion business loan*									
No	Ref			Ref			Ref		
Yes	1.370	1.201	1.562	1.322	1.193	1.465***	1.349	1.127	1.615***
*Respondent use mobile phone*									
No	Ref			Ref			Ref		
Yes	0.716	0.684	0.750***	0.580	0.560	0.600***	0.682	0.640	0.725***
*Able to use SMS*									
No	Ref			Ref			Ref		
Yes	0.661	0.570	0.767	0.669	0.598	0.748***	0.737	0.600	0.906*

***P < 0.001; *P < 0.05

Moreover, the women who go to the cinema or theatre at least once a month are more likely to be victims of physical violence (OR: 1.17, CI 1.007–1.35). The working women are more likely to be exposed to all the three types of IPV, as compared to their non-working counterparts. In addition, the respondents who have knowledge of business loans for women are more likely to experience both physical (OR: 1.63, CI 1.52–1.74) and sexual violence (OR: 1.61, CI 1.44–1.80). Similarly, the women who have taken start-up or expansion business loan are more likely to be victims of both physical and sexual violence. However, the respondents who use mobile phones are less likely to experience IPV (emotional violence; OR: 0.72, CI 0.68–0.75, physical violence; OR: 0.58, CI 0.56–0.60, sexual violence; OR: 0.68, CI 0.64–0.73) as compared to the non-users. Similarly, when compared to the women who are unable to use SMS, the women who are able to use SMS are less likely to be victims of IPV (Table 3).

## Discussion

Domestic violence is one of the most frequently occurring forms of violence against women, where the perpetrators are usually their husbands or other intimate partners [25]. The present national representative found that 13% women in India experienced emotional violence, 28% physical violence and 7% sexual violence performed by their husbands which were a bit lower than the previous IPV prevalence from 2005 to 2006 [25]. From these statistics, it is evident that the prevalence of IPV against women in India has slightly decreased.

The present study has demonstrated certain demographic and socio-economic indicators as well as certain women empowerment variables which act as potential risk factors or protective factors for occurrence of IPV against women. A study which was conducted in Eastern India, indicated that the prevalence of emotional and physical violence was slightly higher among the urban women. On the other hand, rural women experienced sexual violence [5]. This is in contradiction to our present study, which revealed that the rural women were more exposed to all the three types of violence than the urban women. The probable reasons for this could be higher unemployment, lower educational status and lower socio-economic status in the rural areas. Similar findings have been denoted in several other previous studies, including the WHO multi-country study on women’s health and domestic violence [1, 2, 17, 30, 31].

The previous study showed that the Muslim women experienced more violence than the Hindu women or the women belonging to other religions [25]. Another study in Bangladesh indicated that the Muslim women were exposed to more violence during pregnancy [32]. Similar findings were reflected in some other studies [17, 33]. However, the present study indicated that in India, the women belonging to Hindu religion were more exposed to physical and sexual violence, as compared to the Muslim women or the women belonging to any other religion. Another study conducted in Nepal advocated that the Hindu women experienced more emotional violence than their non-Hindu counterparts [30]. These variations among countries could be due to the socio-cultural and economic differences as well as the differences in women’s status and empowerment. In addition, the present study also indicated that the women belonging to Scheduled Caste experienced more emotional, physical and sexual violence than among the women belonging to other castes.

Educational status and socio-economic background were found to have significant association with alleviation of risk of IPV among the Indian women. The present study also indicated that the prevalence of all the three types of IPV was lowest among the women having higher education and among those belonging to the richest wealth quintile. This finding is in line with several previous studies worldwide which stated that the increase in household wealth acts as a protective factor against domestic violence [1, 12, 16–18, 30, 33, 34].

Bridging the gap between the genders and achievement of women empowerment are the most important issues towards attainment of the Sustainable Development Goals (SDGs). Empowerment of women would give them social and financial independence, freedom of movement and decision-making power in the family. Moreover, this would ultimately result in use of modern contraception, antenatal care, institutional delivery, and skilled birth attendance, and most importantly it would result in betterment of children’s health. However, there is need for more in-depth research in order to determine the effect of women’s empowerment on IPV [35]. The present study indicated that the women who did not read newspapers or magazines and those who did not watch television were more exposed to IPV. Similar findings were reflected in the study conducted in Nepal, which stated that exposure of the women to social media, such as radio and television reduced the prevalence of violence against them [30].

The present study also revealed that the women who went to the cinema or theatre at least once a month were more likely to experience physical violence. In addition, the working women respondents were more likely to be exposed to all the three types of violence, than the non-working women. A similar study, which was conducted in India about 10 years back, reflected similar findings [25]. So it is evident that there is not much change in the scenario of IPV against women in India, even after 10 years. Economic independence of women is unable to ensure their protection against domestic violence. This is in line with several other studies [12, 18, 34, 36]. This goes against the “sociological and feminist theories” which state that economic empowerment of women, through education and employment, tend to reduce the prevalence of violence against them [19]. However, a study conducted in Uganda found that economic empowerment of women, in the form of employment reduced their risk of being abused by their husbands or other intimate partners [16]. This variation among the countries could result from the differences in women’s status and socio-cultural norms in different settings. Most often, due to the fear of social stigmatization, women are not ready to speak out or seek help, because of which these crimes often go unreported.

A study conducted in Bangladesh advocated that the inclusion of women in microfinance program did not necessarily result in alleviation of IPV against them. Moreover, the women having formal education, when included in the microfinance program, experienced higher levels of violence at home [17]. The current study indicated that having knowledge of business loans for women and being recipient of such start-up or expansion business loans were significantly associated with violence against women. Being important predictors for IPV, these respondents were more likely to be abused and victimized by their husbands or intimate partners. The probable reason behind this could be the patriarchal mindset of the society that usually exists in low and middle income countries. Because of the inherent gender bias, the men are thought of as the sole bread-winners of the family, which gives rise to male dominance and lowers the status of women, both in the family and in the society. As a result, the men wish to manage the loans single-handedly in the family and any interference or contribution by women is perceived as a threat to their ego. In addition, the victims of domestic violence, usually belonging to the poor families, tend to seek economic independence, in the form of employment or independent business loans. Therefore, it can be concluded that this is an effect of IPV, rather than the cause of it.

Several studies, conducted previously, advocated that the use of electronic and mobile technologies is an important step towards patient empowerment [37–39]. Similarly, studies have also stated that Information and Communication Technologies or ICT has an important role to play in the achievement of gender equity as well as women’s empowerment, although it might amplify the existing inequity, because of lower usage of digital technologies by women [40]. However, to the best of the authors’ knowledge, there has been no previous study to determine that electronic or digital empowerment of women acts as a protective factor against IPV. The current study indicated that the respondents who used mobile phones and SMS facility were less likely to experience IPV. The probable reason could be that, by using mobile phones and SMS, they could stay connected with their friends and family, and could seek help when necessary. A previous study conducted in India found that about 24% of the victims of violence sought help from any source, but mostly from informal sources [20].

Oftentimes, women tend to accept the violence and abuse inflicted on them silently, both due to the fear of social stigma and the normalization of IPV in male-dominated society [4, 9, 41]. However, the advent of ICT has helped women to break this cycle to some extent. This is in contradiction to another study conducted in the southern United States, which revealed that the increasing usage of ICT, such as, mobile phones, internet and social networking sites could exacerbate the problems of stalking, cyber violence and harassment faced by the domestic violence survivors. After leaving abusive relationships, women usually face a multitude of problems, including financial crunch, single motherhood etc. and the digital technologies just add to the burden [42].

The present study indicated that the women who had no education but used mobile phones were more exposed to both emotional and sexual violence, and slightly less exposed to physical violence by their husbands or other intimate partners. Therefore, it can be concluded that education is an essential factor towards prevention of IPV. Only electronic empowerment, without the provision of education cannot bring about a considerable change in the prevalence of IPV against women in India.

Appropriate access to mobile phones and SMS facilities might effectively induce economic and social development of women, through the provision of better employment opportunities, quality health care services, and social participation and support. This might ultimately lead to improved help-seeking behavior among the women. Therefore, the women who are electronically empowered are better able to protect themselves from domestic violence. However, due to the “digital divide” which exists within countries, the illiterate women belonging to the lower socio-economic strata have less access and exposure to media as well as mobile phones [40]. As a result, they are unable to achieve electronic empowerment and in the long run, there is no remarkable change in their exposure to IPV. Therefore, it is evident that all these factors, such as, higher education, household wealth, economic empowerment, electronic empowerment and help-seeking behavior of women are interrelated. All these factors acting together have the potential to reduce the prevalence of IPV against women in India.

### Method discussion

The present study has used the nationally representative NFHS-4 data from the 36 member states and union territories of India, permits generalization of the current findings. The cross-sectional study makes it difficult for assigning causality. The present study has only focused on IPV perpetrated by their husbands or intimate partners. However women in India also experience violence by family members, in-laws, their close and distant relatives, friends, acquaintances, neighbors, and by the men in powerful positions, such as, police, soldiers, and political figures and unknown persons which warrant further studies [9, 15, 25, 43, 44]. So further study is warranted in this respect. Longitudinal studies for assigning causality and qualitative studies examining why the use of mobile phone and SMS are protecting the women from being victim of IPV could provide us with better understanding for further policy development.

## Conclusion

Empowerment of women, in terms of education, employment, social support and participation, alone cannot reduce the prevalence of IPV against them. Women with mobile phones are more protected from IPV. Violence against women, being an important public health issue, must be addressed well. With the reduction in prevalence of IPV, there will be betterment of women’s health and as a result, children’s health [6–11]. Empowered women are able to make better decisions about their lives as well as their children’s lives. Women empowerment acts as a protective factor against IPV, when it is combined with gender equity. The current study, using Indian context, has indicated that electronic empowerment by means of mobile devices and appropriate SMS facilities could be used for IPV awareness program, which could be much more beneficial than other programs pursuing from high income countries.

## Data Availability

The study has used NFHS-4 data. Interested readers can contact NFHS-4 for data availability and necessary permission.

## Abbreviations

CEB: Census enumeration blocks
E-empowerment: Electronic empowerment
IPV: Intimate partner violence
LMIC: Low and middle income country
NFHS: National Family Health Surveys
OBC: Other backward classes
PSU: Primary sampling units
SC: Scheduled castes
ST: Scheduled tribes

## References

[1] Abramsky T, Watts CH, Garcia-Moreno C, Devries K, Kiss L, Ellsberg M (2011). What factors are associated with recent intimate partner violence? Findings from the WHO multi-country study on women's health and domestic violence. BMC Public Health.

[2] Pallitto C, Garcia-Moreno C, Jansen H, Heise L, Ellsberg M, Watts CH (2012). Intimate partner violence, abortion, and unintended pregnancy: results from the WHO multi-country study on women's health and domestic violence. Int J Gynecol Obstet.

[3] Dillon G, Hussain R, Loxton D, Rahman S (2013). Mental and physical health and intimate partner violence against women: a review of the literature. Int J Family Med.

[4] Dalal K, Rahman F, Jansson B (2009). Wife abuse in rural Bangladesh. J Biosoc Sci.

[5] Babu BV, Kar SK (2009). Domestic violence against women in eastern India: a population-based study on prevalence and related issues. BMC Public Health.

[6] Kalokhe A, del Rio C, Dunkle K, Stephenson R, Metheny N, Paranjape A (2016). Domestic violence against women in India: a systematic review of a decade of quantitative studies. Glob Public Health.

[7] Bagwell-Gray ME, Messing JT, Baldwin-White A (2015). Intimate partner sexual violence: a review of terms, definitions, and prevalence. Trauma Violence Abuse.

[8] O'Doherty LJ, Hegarty K, Ramsay J, Davidson LL, Feder G, Taft A (2014). Screening women for intimate partner violence in healthcare settings: abridged Cochrane systematic review and meta-analysis. BMJ.

[9] Bradbury-Jones C, Appleton JV, Clark M, Paavilainen E (2017). A profile of gender-based violence research in Europe: findings from a focused mapping review and synthesis. Trauma Violence Abuse.

[10] Violence info. http://apps.who.int/violence-info/.

[11] Bourey C, Williams W, Bernstein EE, Stephenson R (2015). Systematic review of structural interventions for intimate partner violence in low- and middle-income countries: organizing evidence for prevention. BMC Public Health.

[12] Dalal K (2011). Does economic empowerment protect women from intimate partner violence?. J Inj Violence Res.

[13] Kim JC, Watts CH, Hargreaves JR, Ndhlovu LX, Phetla G, Morison LA (2007). Understanding the impact of a microfinance-based intervention on women’s empowerment and the reduction of intimate partner violence in South Africa. Am J Public Health.

[14] Narayana A, Ahamad T (2016). Role of media in accelerating women empowerment. Int J Adv Educ Res.

[15] Gupta J, Falb KL, Lehmann H, Kpebo D, Xuan Z, Hossain M (2013). Gender norms and economic empowerment intervention to reduce intimate partner violence against women in rural Côte d’Ivoire: a randomized controlled pilot study. BMC Int Health Hum Rights.

[16] Kwagala B, Wandera SO, Ndugga P, Kabagenyi A (2013). Empowerment, partner’s behaviours and intimate partner physical violence among married women in Uganda. BMC Public Health.

[17] Dalal K, Dahlström Ö, Timpka T (2013). Interactions between microfinance programmes and non-economic empowerment of women associated with intimate partner violence in Bangladesh: a cross-sectional study. BMJ Open.

[18] Vyas S, Watts C (2009). How does economic empowerment affect women's risk of intimate partner violence in low and middle income countries? A systematic review of published evidence. J Int Dev.

[19] Heise L. What works to prevent partner violence? An evidence overview. Working paper. STRIVE Research Consortium. London School of Hygiene and Tropical Medicine, London. 2011.

[20] Rowan K, Mumford E, Clark C (2015). Is women’s empowerment associated with help-seeking for spousal violence in India?. J Interpers Violence.

[21] Sanawar S, Islam M, Majumder S, Misu F (2018). Women’s empowerment and intimate partner violence in Bangladesh: investigating the complex relationship. J Biosoc Sci.

[22] Eleonora G, Helmut R. Female empowerment and male backlash. CESifo Working Paper, No. 7009. Center for Economic Studies and Ifo Institute (CESifo), Munich. 2018.

[23] World Health Organization (2002). World report on violence and health.

[24] Rowland J (1997). Questioning empowerment: working with women in Honduras.

[25] Dalal K, Lindqvist K (2010). A national study of the prevalence and correlates of domestic violence among women in India. Asia Pac J Public Health.

[26] National Family Health Survey. http://rchiips.org/NFHS/sub_report.shtml. Accessed 12 March 2020.

[27] Kishor S, Johnson K (2004). Profiling domestic violence: a multi-country study.

[28] Strauss MA, Strauss MA, Gelles RJ (1990). Measuring intra-family conflict and violence: the conflict tactics (CT) scales. Physical violence in American families: risk factors and adaptations to violence in 8145 families.

[29] Rutstein SO, Johnson K. The DHS wealth index. DHS Comparative Reports no. 6. Calverton, MD: ORC Macro, 2004.

[30] Dalal K, Wang S, Svanström L (2014). High intimate partner violence against women in Nepal: an analysis through individual, empowerment, family and societal level factors. J Res Health Sci.

[31] Rizvi N, Feroz A, Pervez S, Oyebode O (2019). Prevalence and factors associated with violence against women in Pakistan. J Women’s Health Gynecol.

[32] Naved R, Persson L (2008). Factors associated with physical spousal abuse of women during pregnancy in Bangladesh. Int Fam Plan Perspect.

[33] Koenig M, Ahmed S, Hossain M, Mozumder A (2003). Women's status and domestic violence in rural Bangladesh: individual and community-level effects. Demography.

[34] Rahman M, Hoque M, Makinoda S (2011). Intimate partner violence against women: is women empowerment a reducing factor? A study from a national Bangladeshi sample. J Family Violence.

[35] Ewerling F, Lynch J, Victora C, van Eerdewijk A, Tyszler M, Barros A (2017). The SWPER index for women's empowerment in Africa: development and validation of an index based on survey data. Lancet Glob Health.

[36] Rocca C, Rathod S, Falle T, Pande R, Krishnan S (2008). Challenging assumptions about women's empowerment: social and economic resources and domestic violence among young married women in urban South India. Int J Epidemiol.

[37] Risling T, Martinez J, Young J, Thorp-Froslie N (2017). Evaluating patient empowerment in association with eHealth technology: scoping review. J Med Internet Res.

[38] Calvillo J, Román I, Roa L (2013). How technology is empowering patients? A literature review. Health Expect.

[39] Burr C, Morley J. Empowerment or engagement? Digital health technologies for mental healthcare. SSRN Electronic J. 2019.

[40] Gender equality and empowerment of women through ICT. New York, NY: United Nations, Division for the Advancement of Women, Dept. of Economic and Social Affairs; 2005.

[41] Zhu Y, Dalal K (2009). Childhood exposure to domestic violence and attitude towards wife beating in adult life: a study of men in India. J Biosoc Sci.

[42] Dimond J, Fiesler C, Bruckman A (2011). Domestic violence and information communication technologies. Interact Comput.

[43] Guruge S, Roche B, Catallo C (2012). Violence against women: an exploration of the physical and mental health trends among immigrant and refugee women in Canada. Nutr Res Pract.

[44] Ahmed-Ghosh H (2004). Chattels of society: domestic violence in India. Violence Against Women.

